# Fracture at Elbow Joint, with Cases

**Published:** 1891-01

**Authors:** Andrew Boyd

**Affiliations:** Scottboro, Ala.


### FRACTURE AT ELBOW JOINT—WITH CASES.

BY ANDREW BOYD, M. D., SCOTTBORO, ALA.

Mr. President, and Members of the Tri-State Medical Association :

Our Secretary has again kindly asked me to contribute my mite from Alabama to the Association, and it being almost my first love, outside of my own State Association, I shall ever do what I can for its success and aggrandizement.

It is my purpose in this paper to report three cases of fracture at Elbow Joint, and their treatment. I am not able to theorize on them any, and shall simply give you a statement of the fractures as I met them in country practice, and how I treated them.

Nothing can be more gratifying to a surgeon than the skillful management and good results of his treatment in all fractures, and more especially those at the elbow ; and nothing can be more discouraging than a bad result. A dead monument is bad enough, but a walking one is much worse. In all other departments of medicine, and I may say surgery, kind nature, with her mysterious hand covers up, and bridges over the bad work of the unskillful, but in this she makes the light more glaring, and the eye more clear. We cannot miss our diagnosis and treatment here, and leave nature to work out the success ; the surgeon and nature must be copartners.

I believe now, that after reduction there are but two methods of treatment of fracture at elbow joint; the extended or straight, and the flexed position. My cases will represent both methods, small though they may be, they will show as far as they go, which is right and which is wrong. During a year's residence in the city hospital at Baltimore I never saw a fracture at elbow treated only in flex position, and during my life I never saw my father treat a case any other way, and I remember with him and in the hospital success was the rule in all cases.

A person that has ideas engrafted in to him by his experience and by those in whom he has great confidence, is difficult to change. It is hard to displace these ideas, but if it can be shown by the surgeons of this wise body that another way is better than the one I adopted, I will gladly accept it.

I will now proceed to give you the cases as they occurred, and as reported by me at a meeting of our county Society.

Case 1. Frank H, age seven years, July 10, 1889. On the day before he was riding a horse, fell off backward, left arm extended; falling on hand, when I saw him, 20 hours after accident, found the left elbow very much swollen, and echymosed, no crepitation, fixed pain at the elbow, the limb helpless. On account of great swelling I could not make out the deformity, but could find the four points above the joint; the two condyles, olecranon process, but on further examination I found that in forced flexion the pain was greatly increased and that he was unable to flex or pronate arm at all. My attention was directed to the internal condyle, and I found here a separation. The internal condyle. I reduced it and flexed the arm less than a right angle, and pronated until back of hand was uppermost; this relaxed the flexors and pronator radii-teres. I put the arm up in a common padded paste board splint, moulded to the arm, the splint being less than a right angle, and extending three inches below the axilla to the finger tips. Seven days afterward saw him again, swelling and echymosis nearly gone, little pain and little deformity, did not disturb the position of the arm, put the arm up in a liquid glass apparatus, allowed to remain fifteen days, making twenty two days from time of injury; took this off, found some little deformity, but more from callous than anything else, arm and fore-arm atrophied some, could flex to sixty-five degrees and extend to one hundred and thirty-five, used passive motion, and the next day presented him with a whip and told him to pop it loud and long with the left hand. He has a perfect elbow to day, with very slight deformity; but what does this amount to compared to anchylosis. Has complete flexion and one hundred and seventy-five degrees extension.

Case 2.—Sem C. aged thirty-two years. On the night of November 18th, his horse threw him, falling on right arm in forced extension, saw him fifteen hours after injury, found the right elbow very much swollen and ecchymosed, fixed pain in joint, fore-arm at right angles or nearly so, with arm immovable; alecranon process very prominent and tilted back about an inch with a corresponding depression in front of olecranon and points of condyles could not be found. The diagnosis was made—a backward dislocation of ulna. He was very nervous with a temperature of one hundred one and one half. I gave him half a grain of morphine hypodermically and reduced the dislocation with little trouble. In this manipulation I found crepitation and a fixed pain over the internal condyle; after reducing the dislocation he had little use of arm—he could neither flex nor pronate it; pronation gave him great pain. I found he had also a fracture of the internal condyle; put the fracture in best position possible, and put the arm up in a right angle pasteboard splint, fore-arm midway between pronation and supination; twelve days afterward took this temporary splint off, echymosis and swelling still present, did not disturb position whatever, and put it up in liquid glass, this was allowed to remain fourteen days, twenty-six days from time of injury—the dressing had caused no disturbance, and the following condition was found on its removal. There was considerable thickening on and about the inner aspect of the elbow, this obscured accurate examination of bone parts. The circumference of arm at its middle was one and one half inches smaller, at the elbow one inch larger on the afflicted side than at same point about its fellow. The patient could flex arm to seventy-five degrees and extend to one hundred and thirty-five degrees, pronate and supinate slightly. Movement at joint caused no pain. He was directed to use passive motion constantly through the day and at night to employ massage and poultice. Thirty days after removal of splint, patient could completely flex arm and extend to one hundred and sixty-five degrees, pronation and supination improved accordingly.

Six months afterward no deformity only on close inspection; circumference at albow and at arm and fore-arm very little difference. Complete flexion and one hundred and eighty-five degrees extension, little interference with pronation.

These now, gentlemen, are two cases treated in the flex position, and the results are very gratifying both to me and to the patients. In either case no one would ever suspect from any movement they used, they had had a fracture at elbow joint. Dr. Allice in a paper read before New Jersey State Association extolled the extended position as *the treatment* in all fractures at elbow joint, and stated that Profs. Gross and Agnew and Dr. Packard had adopted this plan and so taught their classes, but I cannot find it in their text books. The only reason he gives that has any weight, it seems to me, and that which he lays the most stress upon, is the deformity you have after treatment in the flex position, which he says you do not have after the extended. This may be true, but oh, how much better it is to have a little deformity than a straight anchylosed arm! And then in either case we run a risk of anchylosis, and how much better to have a flexed one than a straight one.

I shall now report the third case, which I saw in consultation fifteen days after the receipt of injury.

Grider D. aged twelve years, March 15th, 1890. Horse ran away with him, threw him against a tree, producing a fracture of femur, upper third—he fell on his hand, arm in forced extension; producing fracture of the internal condyle without dislocation. The fracture was reduced by Dr. D. and limb put up in straight splint. Fifteen days after this his condition I found—arm at middle three-quarters of an inch smaller, fore-arm at middle half an inch smaller, the inner aspect of elbow thickened and echymosed and one and one-fourth inches larger than fellow. He could supinate and pronate very well, but under an anaesthetic I could not flex the arm five degrees. The little fellow had already received a great shock from the resetting of the femur, but I advised the breaking up of the adhesions, and putting the arm at right angles, but the family and the doctor, who was a brother to the patient, objected. We put the arm up in a starch bandage and left to nature and at the end of twenty days we found nature to do as she always does in such cases—nothing, but to show us up in the glaring light of negligence and gave us a walking monument to point the finger of scorn at us in years to come. To-day that boy has a beautiful elbow. Oh! yes, it could adorn the ball room dress of a beautiful young lady with "low neck and short sleeves;" but the movement of flexion and extension, which some day he will surely miss, when he is compelled to earn his bread by hard strokes, is gone.

He also has very good pronation and supination, but how little use belonging to the stick it does.

Now, gentlemen, I have given you these cases as they occurred, and small though they may be, they show their value pro and con. To summarize the rational reasons why I think the flex position is best. First, in all cases we fear anchylosis, and it is much better to have a flexed anchylosed arm than a straight one. The comparative use in each is apparent.

Second.—When the splints remain twenty-five to thirty days the arm is atrophied and almost immovable—it is here more easy to overcome flexor muscles than extensors. A patient can with more ease extend an arm than flex it.

I can see but one good result that can be obtained in the extended position, and that is less deformity, and how small this is, compared with the good and useful results obtained in the flex.

The true intent of surgery is not to beautify and mould for cupid's eye at the expense of usefulness.

